# Evaluating the Efficacy of Platelet-Rich Plasma in Treating Primary Knee Osteoarthritis: A Prospective Interventional Study

**DOI:** 10.7759/cureus.71415

**Published:** 2024-10-14

**Authors:** Prant Gupta, Akash Jamra, Shashank Prakash, Sameer Gupta, Ajay Bharti

**Affiliations:** 1 Orthopaedics, Gajra Raja Medical College, Gwalior, Gwalior, IND; 2 Orthopaedics, All India Institute of Medical Sciences, Gorakhpur, Gorakhpur, IND; 3 Orthopaedics and Traumatology, Gajra Raja Medical College, Gwalior, Gwalior, IND; 4 Orthopaedic Surgery, All India Institute of Medical Sciences, Gorakhpur, Gorakhpur, IND

**Keywords:** biological therapy, knee osteoarthritis (koa), platelet-rich plasma (prp), vas scores, womac osteoarthritis index

## Abstract

Introduction: Knee osteoarthritis (OA) is a prevalent degenerative joint disorder causing pain, stiffness, and reduced function, significantly impacting the quality of life. Current treatments mainly provide symptomatic relief, with limited efficacy in halting disease progression. Platelet-rich plasma (PRP), a biological therapy rich in growth factors, has gained attention as a potential treatment for knee OA due to its regenerative properties. This study evaluates the efficacy of PRP in managing primary knee OA.

Methodology: This prospective interventional study included 100 patients diagnosed with primary knee OA, categorized using the Kellgren-Lawrence grading scale. Leukocyte-reduced PRP was prepared using the double-spin method and injected into the knee joint. The efficacy of PRP was assessed using the Western Ontario and McMaster Universities Arthritis (WOMAC) Index and Visual Analogue Scale (VAS) pain scores at six weeks, three months, and six months post-injection. Statistical analysis was performed using SPSS version 25.0, with significance set at p<0.05.

Results: Significant improvements in the WOMAC and VAS scores were observed at all-time points post-PRP injection. The WOMAC score decreased from a baseline of 81.06 to 63.52 at six months (p < 0.001), and the VAS score reduced from 7.53 to 3.09 (p < 0.001). PRP was more effective in patients with lower body mass index (BMI) and less severe OA (Grades 1 and 2). Adverse events were mild, with 18% reporting mild pain or swelling.

Conclusion: PRP therapy significantly improves pain and function in patients with primary knee OA, particularly in early-stage disease. The treatment is generally safe, with minor adverse effects. PRP presents a promising non-surgical option, especially for those seeking to delay or avoid knee arthroplasty.

## Introduction

Osteoarthritis (OA) is a degenerative joint disease characterized by focal deterioration of hyaline cartilage due to an imbalance between its regeneration and breakdown, most commonly affecting the knees, hips, spine, and hands [1,2]. In 2019, the global number of people living with OA reached approximately 528 million, marking a 113% increase compared to figures from 1990 [2]. Among these, knee OA is the most common affecting 365 million people worldwide, significantly impacting their quality of life due to pain, stiffness, and loss of function [1,2]. This increasing prevalence is attributed to aging populations, rising obesity rates, body mass index (BMI), and lifestyle factors. It ranks as the second most common rheumatologic issue and the leading joint disease in India, affecting 22-39% of the population [1]. Women are more commonly affected, with prevalence rising sharply with age; symptoms are present in 45% of women over 65 years, with radiological evidence in 70% [1]. Managing knee OA is challenging, as current treatments mainly offer symptomatic relief without halting disease progression and can have side effects. While non-surgical options like weight management, physical therapy, non-steroidal anti-inflammatory drugs (NSAIDs), and corticosteroid injections provide temporary relief, advanced cases often require surgical interventions such as total knee arthroplasty, which come with risks and are not suitable for all patients [3].

In recent years, there has been growing interest in biological therapies that aim to modify the disease process in OA. Among these, platelet-rich plasma (PRP) has emerged as a promising treatment modality. PRP is an autologous blood product that is rich in growth factors and cytokines, which play a crucial role in tissue repair and regeneration [4]. The application of PRP in orthopedic medicine, particularly in the treatment of knee OA, has gained traction due to its potential to stimulate cartilage repair, reduce inflammation, and modulate the catabolic processes involved in OA [5]. The underlying mechanism of PRP in knee OA is thought to involve the delivery of a high concentration of platelets to the affected area, which in turn release growth factors such as transforming growth factor-beta (TGF-β), platelet-derived growth factor (PDGF), and vascular endothelial growth factor (VEGF). These growth factors are believed to promote the regeneration of damaged cartilage, reduce synovial inflammation, and improve joint function [6]. Several studies have reported positive outcomes with PRP injections, including pain reduction, improved joint function, and increased patient satisfaction [7-14]. However, the results have been variable. The present study aims to address this gap in the literature by conducting a prospective study to assess the efficacy of PRP in patients with primary knee OA. This study hopes to contribute to improve the overall management of knee OA.

## Materials and methods

This prospective interventional study was conducted in Gajra Raja Medical College, Gwalior, a government medical college and hospital of Central India, from September 2022 to June 2024 (21 months), following approval from the Institutional Ethics Committee (IEC no.: 67/IEC-GRMC/2022).

Patient selection and grouping

It involved a prospective analysis of 100 patients, who were diagnosed with primary knee OA and attended the department's outpatient clinic. Patients were included in the study if they were classified as grade 0 to 4 on the Kellgren-Lawrence grading scale or grade 1 to 4 on the Ahlback scale and provided informed written consent. Patients who were immunosuppressed; had secondary OA, connective tissue disorders, inflammatory joint disorders, hemoglobin levels below 10 mg%, bone tumors, metabolic bone diseases, and coexisting backaches; or had received steroid injections within the past six months were excluded.

Sample size calculation

The calculation of sample size was done using G*Power software 3.1.9.4 version (Universität Düsseldorf: Psychologie, Germany) with a 95% confidence interval and 90% power for using dependent t-test at effect size 0.358 [12]. Hence, the total sample size was calculated to be 85. However, it was decided to include 100 participants to account for potential loss to follow-up and to improve the robustness of the findings. By increasing the sample size, we aim to enhance the generalizability of the results and ensure that the study remains adequately powered, even if some participants are unable to complete the study or if there is variability in response to the PRP therapy.

PRP preparation and injection protocol

The leukocyte-reduced PRP was prepared using the double-spin method. A volume of 30-50 ml of whole blood was withdrawn from each patient using a 22-gauge needle, and the blood was equally divided into three portions, each of which was placed in a pre-filled test tube containing 1 ml of 3.8% sodium citrate solution. The blood was subjected to centrifugation at two stages: first at 1200 rpm for 10 minutes, followed by a second centrifugation at 2000 rpm for 10 minutes. This process separated the blood into three distinct layers: platelet-poor plasma (PPP), PRP, and red blood cells (RBCs). The PRP was then carefully extracted from the middle layer.

**Figure 1:**
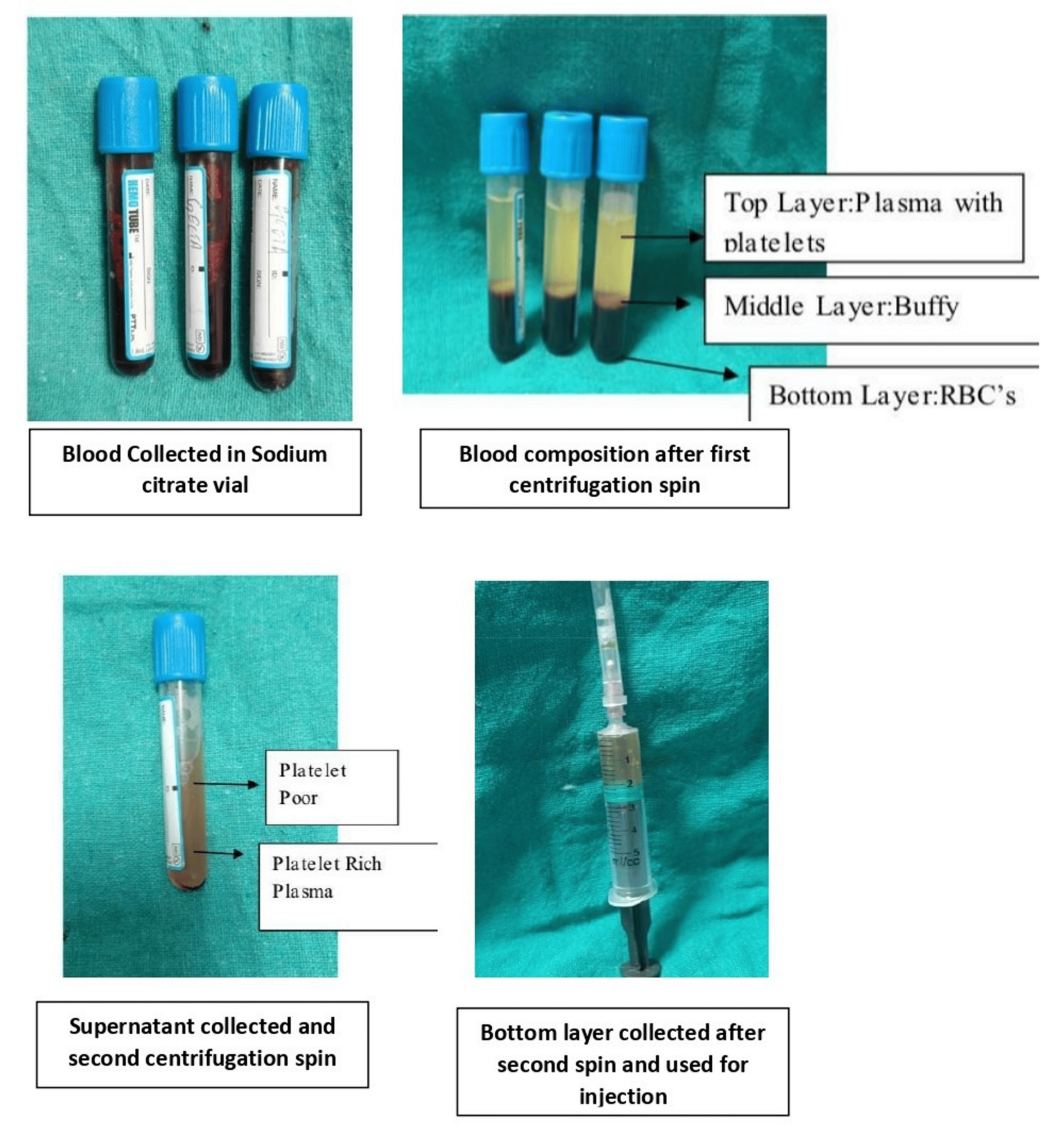
Preparation of leukocyte-reduced platelet-rich plasma (PRP)

Subsequently, the PRP was injected into the suprapatellar pouch or the joint cavity of the knee under sterile, aseptic conditions in an operating theatre. Only a single injection per patient was given. Patients were observed for two hours post-injection and were advised to avoid NSAIDs for two days before and after the procedure. In cases of febrile illness or discomfort due to pain, paracetamol (500 mg) was permitted.

Assessment and follow-up

The patients were monitored at six weeks, three months, and six months post-injection to assess the efficacy of the treatment, using the Western Ontario and McMaster Universities (WOMAC) Index, which evaluates pain, stiffness, and physical function. In addition, the Visual Analog Scale (VAS) was employed to measure pain levels before and after the PRP injections.

Statistical analysis

It was conducted using IBM SPSS Statistics, version 25.0 (trial version, IBM Corp., Armonk, NY). Continuous variables were expressed as mean ± standard deviation and categorical variables as frequencies and percentages. Data visualization was performed using figures and tables. Repeated-measures ANOVA was used to analyze changes in WOMAC and VAS scores over time, while one-way ANOVA assessed differences in WOMAC score improvement across Kellgren-Lawrence grades. An independent t-test was applied to compare improvements between male and female participants. Pearson correlation was used to evaluate the relationship between BMI and WOMAC score improvement. A p-value of less than 0.05 was considered statistically significant with a 95% confidence level.

## Results

Our study assessed the efficacy of PRP injections in 100 participants with primary knee OA. The results of the study are presented below.

Patient demographics

The study included 100 participants, in which the mean age was 50 years (±7.45), with a gender distribution of 21% male and 79% female. The mean BMI was 25.62 kg/m² (±3.03). The participants were categorized according to the Kellgren-Lawrence grading scale, with 35% in Grade 1, 29% in Grade 2, 17% in Grade 3, and 19% in Grade 4. The baseline WOMAC score was 81.06 (±2.53), and the baseline VAS pain score was 7.53 (±0.502). Table 1 provides an overview of the baseline characteristics of the study population.

**Table 1:** Baseline characteristics of the study population WOMAC: Western Ontario and McMaster Universities Arthritis

Variables		Values
Total participants		100
Age (in years) (Mean±S.D.)		50±7.45
Gender	Male, n (%)	21 (21%)
	Female, n (%)	79 (79%)
BMI (kg/m^2^) (Mean±S.D.)		25.62±3.03
Kellgren-Lawrence Grade	Grade 1 n (%)	35 (35%)
	Grade 2 n (%)	29 (29%)
	Grade 3 n (%)	17 (17%)
	Grade 4 n (%)	19 (19%)
Baseline WOMAC Score (Mean±S.D.)		81.06±2.53
Baseline WOMAC Score (Mean±S.D.)		7.53±0.502

WOMAC and VAS pain scores at various intervals

The WOMAC and VAS pain scores were assessed at different times after receiving PRP injections. The mean WOMAC score decreased significantly from 81.06 (±2.53) at baseline to 72.94 (±3.75) at six weeks, 68.75 (±4.05) at three months, and 63.52 (±2.99) at six months (p < 0.001 for all time points). Similarly, the VAS pain score also showed a significant reduction from 7.53 (±0.50) at baseline to 6.38 (±0.72) at six weeks, 5.45 (±0.78) at three months, and 3.09 (±0.79) at six months (p < 0.001 for all time points). Table 2 shows the changes in WOMAC and VAS pain scores at various intervals after PRP injections. A paired "t" test was applied. P value <0.05 was taken as statistically significant.

**Table 2:** Changes in WOMAC and VAS Pain scores at various intervals after PRP injections among the study population Repeated measures ANOVA applied for p-values. P-value <0.05 was taken as statistically significant. WOMAC: Western Ontario and McMaster Universities Arthritis, VAS: Visual Analogue Scale, ANOVA: analysis of variance, PRP: platelet-rich plasma

Outcome measure	Baseline (Mean±S.D.)	Six weeks (Mean±S.D.)	Three months (Mean±S.D.)	Six months (Mean±S.D.)
WOMAC score	81.06±2.53	72.94±3.75*	68.75±4.05*	63.52±2.99*
p-value	-	<0.001	<0.001	<0.001
VAS Pain Score	7.53±0.50	6.38±0.72*	5.45±0.78*	3.09±0.79*
p-value	-	<0.001	<0.001	<0.001

Significant improvements were observed across all grades with percentage improvements at six months being -23.98% for Grade 1 and Grade 2, -17.85% for Grade 3, and -17.27% for Grade 4. The p-value for the changes across all grades was <0.0001 at six weeks, three months, and six months, indicating statistical significance. Table 3 evaluates the association and percentage improvement of WOMAC scores stratified by the Kellgren-Lawrence grading of knee OA severity.

**Table 3:** Association and percentage improvement of WOMAC score stratified by Kellgren-Lawrence grading of knee osteoarthritis severity of study population 1. One-way ANOVA was applied. P-value <0.05 was considered as statistically significant. 2. A paired "t" test was applied. P-value of <0.05 was considered as statistically significant. WOMAC: Western Ontario and McMaster Universities Arthritis, ANOVA: analysis of variance

Kellgren-Lawrence Grade	WOMAC score			
	Baseline (Mean±S.D.)	Six weeks (Mean±S.D.)	Three months (Mean±S.D.)	Six months (Mean±S.D.)
Grade 1 (n = 35)	81.11±2.79	71.06±3.88	65.83±1.70	61.66±1.34
Grade 2 (n = 29)	80.97±2.82	71.62±3.48	66.03±0.94	61.62±1.08
Grade 3 (n = 17)	81.41±2.03	75.59±1.27	74.06±1.39	66.88±2.17
Grade 4 (n = 19)	80.79±2.09	76.05±1.43	73.53±1.54	66.84±2.24
p-value^1^	0.900	<0.001*	<0.001*	<0.001*
Grade 1- Percentage improvement		-12.39%	-18.84%	-23.98%
Grade 2- Percentage improvement		-11.55%	-18.45%	-23.98%
Grade 3- Percentage improvement		-7.15%	-9.03%	-17.85%
Grade 4- Percentage improvement		-5.87%	-8.99%	-17.27%
p-value^2^		<0.0001*	<0.0001*	<0.0001*

Table 4 details the association and percentage improvement of WOMAC scores by gender. While both males and females showed improvement, with females showing slightly higher percentage improvements at each time point, the p-values indicated no statistically significant difference between genders (p > 0.05 at all the time points). A paired "t" test was applied. A P-value <0.05 was taken as statistically significant.

**Table 4:** Association and percentage improvement of WOMAC score with gender of the study population A paired "t" test was applied. P-value <0.05 was taken as statistically significant. WOMAC: Western Ontario and McMaster Universities Arthritis

Gender	WOMAC score			
	Baseline (Mean±S.D.)	Six weeks (Mean±S.D.)	Three months (Mean±S.D.)	Six months (Mean±S.D.)
Male (n = 21)	80.78±2.595	72.80±3.943	68.95±4.293	63.80±3.119
Female (n = 79)	82.10±2.047	73.48±2.960	68.00±2.915	62.48±2.250
p-value	0.015*	0.387	0.347	0.081
Male- Percentage improvement		-10.50%	-15.17%	-21.02%
Female- Percentage improvement		-9.88%	-14.64%	-23.90%
p-value		0.464	0.342	0.072

Correlation analysis between the body mass index (BMI) and WOMAC score improvement

Table 5 presents the correlation analysis between the BMI and WOMAC score improvement. A statistically significant negative correlation was observed at all time points, with correlation coefficients of -0.230 at six weeks (p = 0.021), -0.541 at three months (p < 0.001), and -0.496 at six months (p < 0.001), indicating that a higher BMI was associated with less improvement in WOMAC scores. A paired "t" test was applied. P-value <0.05 was taken as statistically significant.

**Table 5:** Correlation analysis between the body mass index (BMI) and WOMAC score improvement Pearson correlation applied for p-values. P-value <0.05 was taken as statistically significant. WOMAC: Western Ontario and McMaster Universities Arthritis

Time point	Correlation Coefficient (r)	p-value
Six weeks	-0.230	0.021*
Three months	-0.541	<0.001*
Six months	-0.496	<0.001*

Adverse events

**Figure 2:**
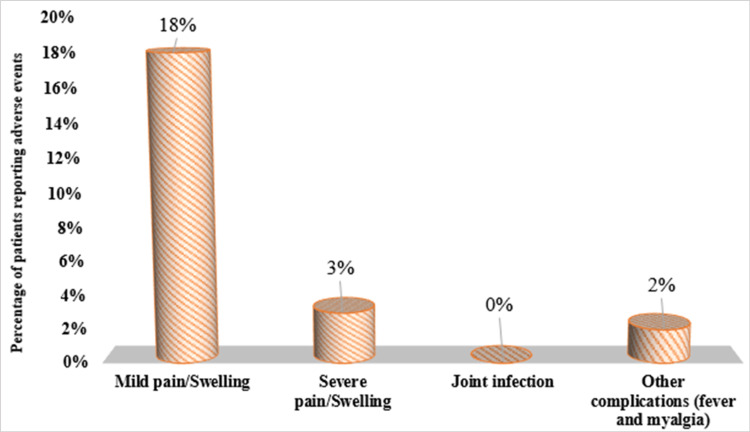
Adverse events after PRP injections among the study population PRP: platelet-rich plasma

Figure 2 depicts the adverse events after PRP injections. Mild pain or swelling was reported by 18% of the participants, severe pain or swelling by 3%, and other complications such as fever and myalgia were reported by 2%. No joint infections were observed.

## Discussion

The present study on the use of PRP in treating primary knee OA involved 100 participants, with a mean age of 50.0 ± 7.45 years. The cohort primarily consisted of older adults, with a higher proportion of females (79%) compared to males (21%), reflecting the known higher prevalence of OA in women. This finding aligns with studies such as Shane Anderson A and Loeser RF (2010) [15], which reported a greater incidence of knee OA in females over the age of 50. Similar trends were observed in other studies, such as those by Muraki S et al. (2009) [16], Zhang Y and Jordan JM (2013) [17], and Srikanth VK et al. (2005) [18], which demonstrated a higher prevalence of radiographic knee OA in elderly women.

Obesity emerged as a significant risk factor for knee OA in the present study. Excess adipose tissue in obese individuals produces hormonal factors that alter cartilage metabolism (leptin system), potentially linking metabolic abnormalities to an increased risk of OA, as suggested by Grazio S and Balen D (2015) [19]. High BMI was found to be significantly associated with knee and hand OA, reinforcing the relationship between obesity and joint health, as per Grotle M et al. (2008) [20]. Occupational activities such as kneeling and squatting were identified as primary risk factors for knee disorders. Reid and Bush (2010) [21] and Zhang Y and Jordan JM (2013) [17] observed that frequent squatting could predispose individuals to knee OA, particularly in elderly populations who reported prolonged squatting during their younger years. OA, although commonly associated with older adults, also affects younger individuals and athletes. Factors like injuries, occupational activities, and obesity contribute to the onset of OA in these populations. Diagnosing OA in younger individuals can be challenging due to their higher pain tolerance. However, treatment approaches for OA do not differ significantly across age groups, focusing on exercise, NSAIDs, braces, and surgical options when necessary [22].

Varying degrees of knee OA severity were exhibited by the participants of this study, categorized using the Kellgren-Lawrence grading system. Most participants had mild (Grades 1 and 2) OA, while others had moderate to severe (Grades 3 and 4) OA. The study found that PRP injections were more effective in participants with less severe OA (Grades 1 and 2). These participants showed greater improvements in WOMAC scores compared to those with more severe OA, although all groups benefited from the treatment over time. Studies by Saraf A et al. (2022) [23] and Huda N et al. (2022) [24] support the findings that PRP therapy can provide short-term pain relief and functional improvement in knee OA, particularly in earlier stages. PRP's potential to slow disease progression makes it a valuable option for delaying or avoiding surgical intervention.

The present study reported the greatest improvement in WOMAC and VAS pain scores six months after PRP injections, although there was a gradual decline in efficacy by that time. The duration of PRP’s effects tends to vary across studies, with many indicating that the maximum efficacy is observed within the first six months after the injection. However, some studies suggest that the benefits can last up to 12 months, albeit with diminishing effects over time [25]. PRP's mechanism of action, involving growth factors and cytokines, contributes to temporary cartilage repair and reduced joint inflammation, explaining the initial improvement in scores. These findings are consistent with Wang YC et al. (2022) [26] and Tang JZ et al. (2020) [13].

Regarding the necessity of repeat injections, several studies have demonstrated that multiple PRP injections, typically administered over weeks, may provide better long-term outcomes compared to a single injection. For example, research has shown that three or more injections yield sustained improvements in knee function and pain relief for up to six months or more​. In our study, while only one injection was administered, patients may benefit from repeat injections depending on their disease severity and initial response [27].

In the present study, the majority of participants (18%) experienced mild pain or swelling at the injection site, a common side effect of intra-articular injections. A smaller proportion (3%) reported severe pain or swelling, but no cases of joint infection were observed, indicating that the procedure was performed under strict aseptic conditions. Two participants (2%) reported fever and myalgia, although these were not detailed in the study's tables. Adverse events such as temporary pain, infection risk, inflammation, and rare allergic reactions were documented, with no permanent adverse effects noted. Studies by Annaniemi JA et al. (2023) [28] and Bansal H et al. (2021) [25] reported prolonged pain and synovitis as common complaints, resolving within a week. While PRP therapy is generally safe, patients should be informed of potential risks and discuss them with their healthcare provider before treatment.

Both male and female participants in the present study showed improvements in WOMAC scores after PRP treatment, with no statistically significant differences between genders. This suggests that PRP treatment is equally effective for male and female knee OA patients, consistent with findings by Evanson JR et al. (2014) [29].

The present study found that participants with lower BMI experienced greater improvements in WOMAC scores following PRP treatment, especially at the three-month follow-up. Lower BMI may enhance PRP's effectiveness due to better accessibility of the target area, healthier tissue quality, reduced mechanical stress, and a more robust healing response. However, obese patients still benefited from PRP injections, although the effects diminished more quickly, increasing their risk for future arthroplasty [30].

Implications for clinical practice

The results of our study demonstrate that PRP therapy significantly improves pain and function in patients with primary knee OA, particularly in those with early-stage disease. This is particularly relevant given the limitations of conventional treatments that primarily offer symptomatic relief without addressing the underlying degenerative processes. Our findings support the incorporation of PRP therapy as a viable non-surgical option for managing knee OA. Clinicians may consider offering PRP to patients who are in the early stages of OA and seek to delay or avoid more invasive procedures like knee arthroplasty. The study highlights that patients with lower BMI and less severe OA (Kellgren-Lawrence Grades 1 and 2) experience more significant benefits from PRP treatment. This information can aid clinicians in selecting appropriate candidates for PRP therapy, optimizing treatment outcomes. By demonstrating the efficacy and safety of PRP, our study encourages a more comprehensive management approach that includes biologic therapies alongside traditional methods such as physical therapy and lifestyle modifications. This multimodal strategy could enhance overall patient satisfaction and improve quality of life. The promising results of our study warrant further investigation into the long-term effects of PRP therapy and its potential role in combination with other treatment modalities. Future research could explore optimal injection protocols, frequency, and patient follow-up strategies. Incorporating these considerations will provide a more holistic view of how our findings can be applied in clinical settings and contribute to the evolving landscape of knee OA management.

Limitations of the study

This study has several limitations. The small sample size and single-center design may limit the generalizability of the findings. The six-month follow-up period is relatively short, potentially overlooking the long-term efficacy and adverse effects of PRP therapy. In addition, the absence of a control group and reliance on subjective outcome measures like the WOMAC Index and VAS pain scores may introduce bias. Lastly, variations in PRP preparation and potential selection bias due to convenience sampling could affect the consistency and applicability of the results.

## Conclusions

This study demonstrates that PRP therapy significantly improves pain and function in patients with primary knee OA, particularly in the early stages of the disease. The greatest benefits were observed within six months post-treatment, with a noticeable reduction in WOMAC and VAS scores. However, patients with higher BMI and more severe OA showed less improvement. While the treatment is generally safe, minor adverse effects were reported. PRP offers a promising non-surgical option, especially for those aiming to delay or avoid knee arthroplasty.

## References

[1] Pal CP, Singh P, Chaturvedi S, Pruthi KK, Vij A (2016). Epidemiology of knee osteoarthritis in India and related factors. Indian J Orthop.

[2] (2024). Osteoarthritis. Osteoarthritis.

[3] Hsu H, Siwiec RM (2024). Knee osteoarthritis. StatPearls [Internet].

[4] Verma R, Kumar S, Garg P, Verma YK (2022). Platelet-rich plasma: a comparative and economical therapy for wound healing and tissue regeneration. Cell Tissue Bank.

[5] Blaga FN, Nutiu AS, Lupsa AO, Ghiurau NA, Vlad SV, Ghitea TC (2024). Exploring platelet-rich plasma therapy for knee osteoarthritis: an in-depth analysis. J Funct Biomater.

[6] Everts P, Onishi K, Jayaram P, Lana JF, Mautner K (2020). Platelet-rich plasma: new performance understandings and therapeutic considerations in 2020. Int J Mol Sci.

[7] Patel S, Dhillon MS, Aggarwal S, Marwaha N, Jain A (2013). Treatment with platelet-rich plasma is more effective than placebo for knee osteoarthritis: a prospective, double-blind, randomized trial. Am J Sports Med.

[8] Kalbkhani M, Dehghani S, Najafpour A, Haddadi N, Mohamad Hossein K (2014). Effects of platelet rich plasma (PRP) in treatment of experimentally induced osteoarthritis in rabbit’s knee joint. Adv Stem Cells.

[9] Gong H, Li K, Xie R (2021). Clinical therapy of platelet-rich plasma vs hyaluronic acid injections in patients with knee osteoarthritis: a systematic review and meta-analysis of randomized double-blind controlled trials. Medicine (Baltimore).

[10] Dai WL, Zhou AG, Zhang H, Zhang J (2017). Efficacy of platelet-rich plasma in the treatment of knee osteoarthritis: a meta-analysis of randomized controlled trials. Arthroscopy.

[11] Kanchanatawan W, Arirachakaran A, Chaijenkij K, Prasathaporn N, Boonard M, Piyapittayanun P, Kongtharvonskul J (2016). Short-term outcomes of platelet-rich plasma injection for treatment of osteoarthritis of the knee. Knee Surg Sports Traumatol Arthrosc.

[12] Moretti L, Maccagnano G, Coviello M, Cassano GD, Franchini A, Laneve A, Moretti B (2022). Platelet-rich plasma injections for knee osteoarthritis treatment: a prospective clinical study. J Clin Med.

[13] Tang JZ, Nie MJ, Zhao JZ, Zhang GC, Zhang Q, Wang B (2020). Platelet-rich plasma versus hyaluronic acid in the treatment of knee osteoarthritis: a meta-analysis. J Orthop Surg Res.

[14] Filardo G, Previtali D, Napoli F, Candrian C, Zaffagnini S, Grassi A (2021). PRP injections for the treatment of knee osteoarthritis: a meta-analysis of randomized controlled trials. Cartilage.

[15] Shane Anderson A, Loeser RF (2010). Why is osteoarthritis an age-related disease?. Best Pract Res Clin Rheumatol.

[16] Muraki S, Oka H, Akune T (2009). Prevalence of radiographic knee osteoarthritis and its association with knee pain in the elderly of Japanese population-based cohorts: the ROAD study. Osteoarthritis Cartilage.

[17] Zhang Y, Jordan JM (2010). Epidemiology of osteoarthritis. Clin Geriatr Med.

[18] Srikanth VK, Fryer JL, Zhai G, Winzenberg TM, Hosmer D, Jones G (2005). A meta-analysis of sex differences prevalence, incidence and severity of osteoarthritis. Osteoarthritis Cartilage.

[19] Grazio S, Balen D (2009). Obesity: risk factor and predictor of osteoarthritis [Article in Crotian]. Lijec Vjesn.

[20] Grotle M, Hagen KB, Natvig B, Dahl FA, Kvien TK (2008). Obesity and osteoarthritis in knee, hip and/or hand: an epidemiological study in the general population with 10 years follow-up. BMC Musculoskelet Disord.

[21] Reid CR, Bush PM, Cummings NH, McMullin DL, Durrani SK (2010). A review of occupational knee disorders. J Occup Rehabil.

[22] Buckwalter JA, Lane NE (1997). Athletics and osteoarthritis. Am J Sports Med.

[23] Saraf A, Hussain A, Bishnoi S, Azam G, Habib H (2022). Serial platelet-rich plasma intra-articular injections in Kellgren and Lawrence grade IV knee joint osteoarthritis: a prospective blinded placebo-controlled interventional study. Indian J Orthop.

[24] Huda N, Islam MS, Bishnoi S, Kumar H, Aggarwal S, Ganai AA (2021). Role of triple injection platelet-rich plasma for osteoarthritis knees: a 2 years follow-up study. Indian J Orthop.

[25] Bansal H, Leon J, Pont JL, Wilson DA, Bansal A, Agarwal D, Preoteasa I (2021). Platelet-rich plasma (PRP) in osteoarthritis (OA) knee: correct dose critical for long term clinical efficacy. Sci Rep.

[26] Wang YC, Lee CL, Chen YJ (2022). Comparing the efficacy of intra-articular single platelet-rich plasma(prp) versus novel crosslinked hyaluronic acid for early-stage knee osteoarthritis: a prospective, double-blind, randomized controlled trial. Medicina (Kaunas).

[27] Huang HY, Hsu CW, Lin GC, Lin HS, Chou YJ, Liou IH, Sun SF (2022). Comparing efficacy of a single intraarticular injection of platelet-rich plasma (PRP) combined with different hyaluronans for knee osteoarthritis: a randomized-controlled clinical trial. BMC Musculoskelet Disord.

[28] Annaniemi JA, Pere J, Giordano S (2023). The effects of platelet-rich plasma injections in different stages of knee osteoarthritis. Eur J Orthop Surg Traumatol.

[29] Evanson JR, Guyton MK, Oliver DL (2014). Gender and age differences in growth factor concentrations from platelet-rich plasma in adults. Mil Med.

[30] Luo P, Xiong Z, Sun W (2020). How to choose platelet-rich plasma or hyaluronic acid for the treatment of knee osteoarthritis in overweight or obese patients: a meta-analysis. Pain Res Manag.

